# Late failure of posterior fixation without bone fusion for vertebral metastases

**DOI:** 10.1590/1413-785220152306151402

**Published:** 2015

**Authors:** Renato Tavares Bellato, William Gemio Jacobsen Teixeira, Alessandro Gonzalez Torelli, Alexandre Fogaça Cristante, Tarcísio Eloy Pessoa de Barros, Olavo Pires de Camargo

**Affiliations:** 1 Universidade de São Paulo, Faculdade de Medicina, Hospital das Clínicas, Institute of Orthopedics and Traumatology, São Paulo, SP, Brazil; 2 Instituto do Câncer do Estado de São Paulo, Spine Group, São Paulo, SP, Brazil

**Keywords:** Spinal neoplasms, Arthrodesis, Spinal fusion, Re-trospective studies

## Abstract

**OBJECTIVE:**

: To verify the frequency of late radiological com-plications in spinal fixation surgeries performed without fu-sion in oncological patients

**METHODS:**

: This is a retrospective analysis analysing failure in cases of non-fused vertebral fixation in an oncology reference hospital between 2009 and 2014. Failure was defined as implant loosening or bre-akage, as well as new angular or translation deformities

**RESULTS:**

: One hundred and five cases were analyzed. The most common site of primary tumor was the breast and the most common place of metastasis was the thoracic spine. The average follow-up was 22.7 months. Nine cases (8%) of failure were reported, with an average time until failure of 9.5 months. The most common failure was implant loosening. No case required further surgery

**CONCLUSION:**

: The occurrence of failure was not different than that reported for fused cases. The time interval until failure was higher than the median of survival of the majority (88%) of cases. **Level of Evidence IV, Therapeutic Study.**

## INTRODUCTION

The spine is the most common site of metastatic bone disease, especially in patients with breast, lung or prostate cancer. 1 In autopsy studies, it can reach up to 90% of cases. In up to 20% of patients, symptoms related to vertebral metastases are the first manifestation of cancer.

Treatment of metastatic disease of the spine aims pain mana-gement, maintenance or recovery of walking ability and neu-rological function, and maintenance of stability and quality of life.^2^ The main methods of treatment are radiotherapy, surgery, chemotherapy, hormonal treatment and immunotherapy, which may be used alone or in combination, according to histology and the patient's clinical condition.

The role of surgery is well established in the treatment of pa-tients with high-grade spinal cord compression due to solid tumor.^2^ ^3^ It is also indicated for patients without spinal cord compression, but with pain due to mechanical instability.^4^ In the presence of instability, with or without spinal cord compres-sion, surgery should be performed with spine fixation to ensure mechanical stability. It can be done via posterior way, anterior way, or combination of both.

Ideally, the fixation of the spine should be associated with bone fusion, in order to avoid late failure of instrumentation.^5^ In order to obtain the fusion, it is important to ensanguine the articular surfaces of the spine. Autograft can be used to achieve con-solidation, with increased surgical time and morbidity at the donor site. When using synthetic bone substitutes, the cost of the procedure is increased. Despite the importance of arthro-desis in other spine diseases, its effectiveness is questionable in the metastatic disease of the spine: bone healing capacity is impaired by the effect of adjuvant radiation therapy, malnu-trition and chemotherapeutic drugs.^6^ ^7^ Considering that survival of patients with spinal metastatic disease is limited,^8^ and that their functional demands is generally reduced, it is possible that consolidation of arthrodesis is not crucial in this patient group. If the rate of mechanical complications related to fixation wi-thout fusion is low, the possibility of using percutaneous fixation systems for spine stabilization is increased, with the potential reduction of surgical morbidity. 9 10

The objective of this study was to describe the rate of mecha-nical complications in the surgical treatment of spinal metas-tases in patients with spinal cord compression by metastatic solid tumors that underwent decompression and fixation with posterior approach without arthrodesis 

## METHODS

This study was approved by the Research Ethics Committee of *Instituto do Câncer do Estado de São Paulo* (ICESP), São Paulo, SP, Brazil, under protocol number NP 228-12.

The study included patients operated between February 2009 and January 2014 at ICESP due to spinal cord compression, radicular compression or spinal instability, submitted to fixation without bone fusion, with or without decompression. Medical records and imaging tests were retrospectively evaluated. Be-cause of the retrospective observational nature of the study, Free and Informed Consent Term was not provided.

Patients with diagnosis of spinal metastasis of solid tumor in the mobile spine confirmed by CT or MRI, aged over 18 years old who underwent surgery exclusively by the posterior approach with fixation without arthrodesis, with or without decompression, were included in the study.

Patients from previous surgery history in the area subjected to surgical treatment, patients with previous surgery made by the anterior approach or combined techniques, and those who died or lost follow-up with less than 30 days were excluded.

The medical records were analyzed in terms of age, gender, location of primary tumor, region of the spine affected (Oc-cipitocervical C0-C1; Cervical C2-C6; Cervicothoracic C7-T1; Thoracic T2-T11; Thoracolumbar T12-L1; Lumbar L2-L5; Lum-bosacral L5-S1), approach, time between fixation and the last X-ray control or diagnosis of mechanical failure and the time be-tween surgery and death or last medical evaluation if still alive. The primary endpoint evaluated was the occurrence of failure of spine fixation defined by the presence of new kyphosis or scoliosis deformity above the fifth, translation between adjacent vertebrae above 3 mm, osteolysis along the screw, loosening or breakage of the implant. The evaluation of fixation integrity was performed with plain radiography and/or CT scan. The need for reoperation in cases of failure was also recorded.

## RESULTS

A total of 140 medical records of patients undergoing fixation via posterior approach in the context of metastatic disease of the spine were reviewed. Most (65%) was admitted for emer-gency surgery due to neurological deficit resulting from spinal cord compression.

Thirty-five patients (25%) were excluded by loss of follow-up time of less than 30 days. Of these, 31 were excluded be-cause they died and four due to loss of follow-up. Thus, the final sample suitable for analysis was of 105 patients.

Among the cases, 51 patients were female and 54 male pa-tients. The mean age was 56.71 years old (±12.4 years). The most common locations of the primary tumor were: breast (26 cases), kidney (21 cases), lung (12 cases) and colorectal can-cer (10 cases). The area most commonly affected was the tho-racic spine (44 cases), followed by thoracolumbar spine (34 cases). The cervicothoracic regions of the spine and lumbar and lumbosacral were also affected, with nine patients each, while the cervical region was less affected, with two cases. The mean follow-up after the procedure was 22.76 months (±9.6 months). The demographics of the study population is shown in Table 1.

**Figure 1:**
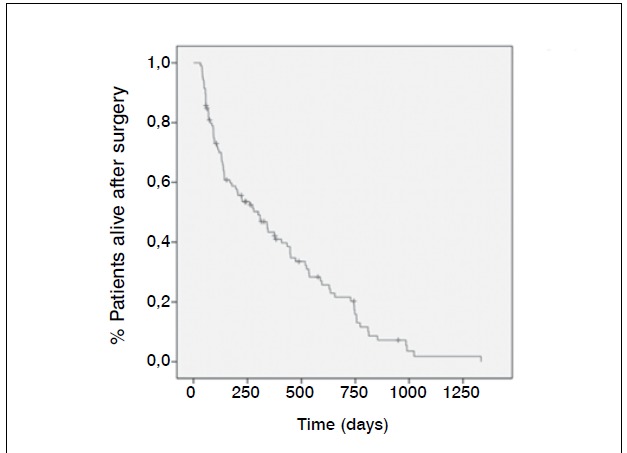
Survival curve (in days) of the studied population after surgery

Throughout the analyzed sample nine mechanical failure events

(8%) were observed, distributed according to data on Tables 2-^5^. The mean time of failure diagnosis was 9.5 months, with survival of the 94% at 12 months and 91% at 24 months. Sur-vival curves of the study population and regarding the surgical procedure are shown in Figures 1 and ^2^.

The most common occurrence was osteolysis along the screws (five cases), followed by avulsion of the proximal or distal lo-cking screws (three cases). (Figures 3 and ^4^) The most com-mon area of ​failure was the thoracic spine (five cases), and there were no failure reports for arthrodesis in the lumbosacral region. There were no patients with neurological deficits related to the mechanical complication, death or last clinical evaluation in this study. Of the mechanical failure cases none was sub-mitted to reoperation.

## DISCUSSION

In 1980, Young et al.^11^ published a prospective randomized trial study comparing surgery by laminectomy followed by radiothe-rapy to radiotherapy alone in the treatment of metastatic spinal cord compression. The authors showed that both methods had similar effectiveness regarding pain, walking ability or sphincter function. From this study, it was recommended that vertebral metastatic lesion from solid tumors were treated with radio-therapy alone. However, surgery, represented by laminectomy, enables only indirect decompression and would not treat the instability potentially caused by tumor lesion or by the extent of decompression.

**Table 1:**
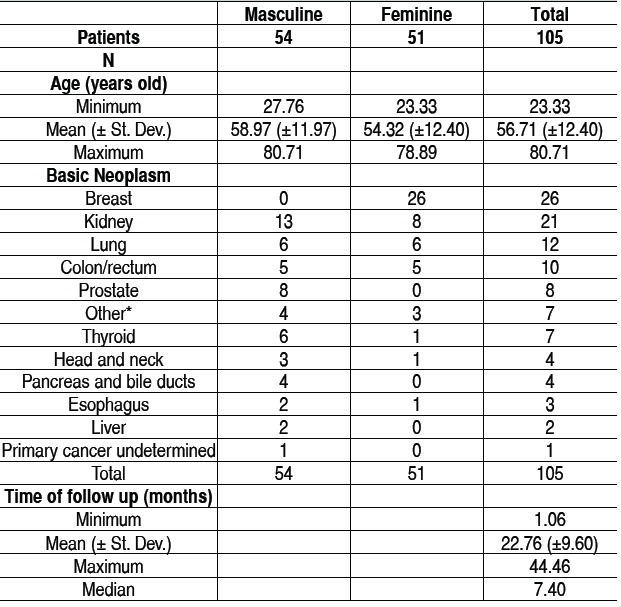
Epidemiological distribution of the patients. Under the asterisk were grouped isolated cases of cancer of the bladder, testicular, cervical, pleura and musculoskeletal system

**Table 2:** Distribution of arthrodesis and failures observed according to the vertebral spine segment.

Region affected	Total	Number of failures
Thoracic	44	5
Thoracolumbar	34	1
Cervicothoracic	9	1
Lumbar	9	1
Lumbosacral	7	0
Cervical	2	1
Total	105	9 (8.57%)

**Table 3:**
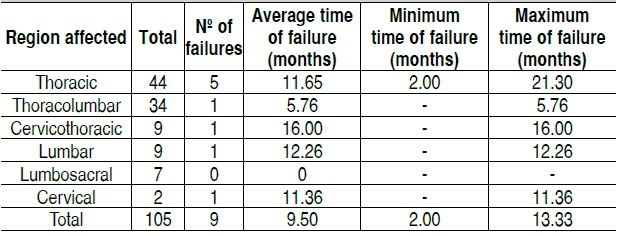
Distribution of failures observed and time of occurrence accor-ding to the vertebral segment affected

**Table 4:**
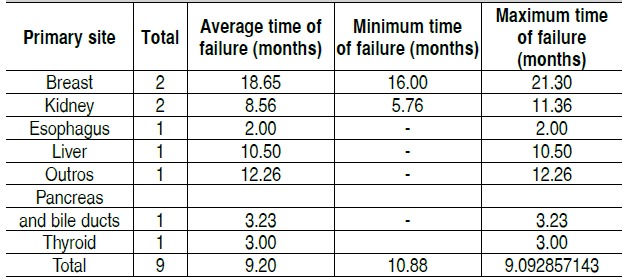
Distribution of failures observed and time of occurrence ac-cording to the primary neoplastic site.

**Table 5:**
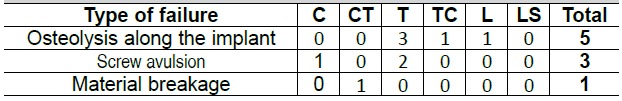
Distribution of failures according to type and vertebral segment.

C: Cervical; CT: Cervicothoracic; T: Thoracic; TC: Thoracolumbar; L: Lumbar; LS: Lumbosacral

With the development of materials for spine reconstruction, several studies have been published showing superior results in the surgical treatment of spinal cord compression due to metastatic solid tumor with fixation and direct decompression of the spinal cord, as compared to laminectomy without fixation.^3^ ^12^ Currently, laminectomy without fixation has been reserved for the treatment of vertebral metastasis in stable spine and lesions solely located in the posterior elements.^13^

Treatments with direct decompression and stabilization chan-ged the results of surgical treatment. Patchel et al.^3^ published in 2005 a clinical trial comparing the circumferential spinal cord decompression and spine fixation to radiotherapy alone. The study was interrupted during mid-term assessment due to the superior effect of surgery in maintaining walking ability. It is believed that the superiority of surgery in the treatment of high grade metastatic spinal cord compression is due to the rapid rate at which decompression is achieved at surgery, besides allowing effective treatment of mechanical instability.

**Figure 2:**
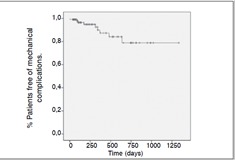
Survival curve without mechanical complications after surgery. Tempo (dias) = Time (days)

**Figure 3:**
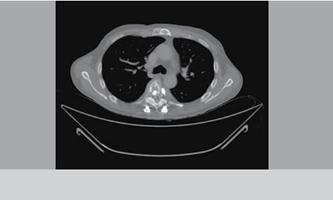
Patient with multiple bone injuries who presented loosening and migration of the implant.

**Figure 4:**
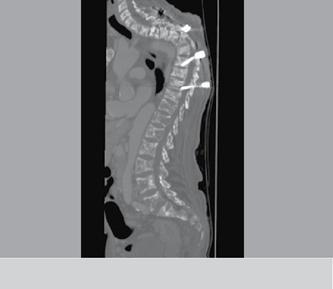
Patient with multiple bone injuries who presented loosening and migration of the implant.

Many efforts have been made to define objective criteria for the assessment of instability in the presence of metastatic spine disease, 14 15, however, there is no appropriate criteria tested in prospective studies. However, it is recognized that the presence of instability is an important factor in deciding between isolated radiotherapy and surgical treatment. The severity of instability also influences the extent of surgical treatment. A limitation of this study is that only patients operated by posterior approach were included. The possible bias of our study resides precisely in the fact that patients included underwent fixation only by one approach, whereas patients with more severe instability are commonly addressed by dual approach.

The importance of column fixation on the treatment of patients with spinal instability is recognized. However, the importance of the successful consolidation of arthrodesis is unknown. The intention to promote fusion increases the surgical time and bleeding potential due to ensanguine the articular surfaces. The use of autologous iliac crest graft promotes potential morbidity at the donor site of the graft,^16^ and the use of bone substitutes increase the cost of the surgical procedure.

The posterior fixation failure rate described elsewhere is 2-8% in 24 months.^17^ ^18^ The total incidence of complications after 24 months in our study population was 8.57%, a value ​similar to the data described in the literature. It is known that the mean survival rate of patients with vertebral metastasis is limited. In this series of cases, the mean survival rate was 22.76 months with a median of 7.4 months. Therefore, 88% of patients had lower survival time than the average time to develop mecha-nical complications. Among the patients who did not develop complications within 24 months of follow-up, there were no new events, until death.

In this study, there was no need to review the patients that pre-sented failure of the fixation system. However, it has not been possible to correlate implant loosening with quality of life of patients due to lack of adequate information in this retrospective study and large sample heterogeneity of patients, resulting in large variability of the evolution of neoplastic disease.

Percutaneous image-guided transpedicular fixation is limited by impossibility to ensanguine articular surfaces and bone grafts. However, in the treatment of metastatic disease, fixation without fusion is feasible. Thus, prospective studies with less invasive techniques are desirable to understand whether there is a re-duction in morbidity and better results could be obtained, as compared to conventional surgery, without adding higher risk of late mechanical complications.

## CONCLUSION

In this case series of patients with vertebral metastases opera-ted with transpedicular fixation exclusively by posterior appro-ach without fusion, the frequency of mechanical complications was 8.57%. However, there was no indication of further surgery for reviewing mechanical problems.
